# Genotype–phenotype correlation in two Polish neonates with alveolar capillary dysplasia

**DOI:** 10.1186/s12887-020-02200-y

**Published:** 2020-06-29

**Authors:** Zuzanna Kozłowska, Zuzanna Owsiańska, Joanna P. Wroblewska, Apolonia Kałużna, Andrzej Marszałek, Yogen Singh, Bartłomiej Mroziński, Qian Liu, Justyna A. Karolak, Paweł Stankiewicz, Gail Deutsch, Marta Szymankiewicz-Bręborowicz, Tomasz Szczapa

**Affiliations:** 1 Department of Neonatology, Neonatal Biophysical Monitoring and Cardiopulmonary Therapies Research Unit, Poznan University of Medical Sciences, Poznan, Poland; 2 Department of Pathology, Poznan University of Medical Sciences and Greater Poland Cancer Center, Poznan, Poland; 3 Department of Neonatology and Paediatric Cardiology, Cambridge University Hospitals NHS Foundation Trust, Cambridge, UK; 4 Department of Pediatric Cardiology and Nephrology, Poznan University of Medical Sciences, Poznan, Poland; 5 Department of Molecular and Human Genetics, Baylor College of Medicine, Houston, TX USA; 6 Chair and Department of Genetics and Pharmaceutical Microbiology, Poznan University of Medical Sciences, Poznan, Poland; 7 Department of Pathology, Seattle Children’s Hospital, Seattle, USA

**Keywords:** Alveolar capillary dysplasia, *FOXF1* mutation, Respiratory failure, Neonate, Pulmonary hypertension

## Abstract

**Background:**

Alveolar capillary dysplasia (ACD) is a rare cause of severe pulmonary hypertension and respiratory failure in neonates. The onset of ACD is usually preceded by a short asymptomatic period. The condition is refractory to all available therapies as it irreversibly affects development of the capillary bed in the lungs. The diagnosis of ACD is based on histopathological evaluation of lung biopsy or autopsy tissue or genetic testing of *FOXF1* on chromosome 16q24.1. Here, we describe the first two Polish patients with ACD confirmed by histopathological and genetic examination.

**Case presentation:**

The patients were term neonates with high Apgar scores in the first minutes of life. They both were diagnosed prenatally with heart defects. Additionally, the first patient presented with omphalocele. The neonate slightly deteriorated around 12^th^ hour of life, but underwent surgical repair of omphalocele followed by mechanical ventilation. Due to further deterioration, therapy included inhaled nitric oxide (iNO), inotropes and surfactant administration. The second patient was treated with prostaglandin E1 since birth due to suspicion of aortic coarctation (CoA). After ruling out CoA in the 3^rd^ day of life, infusion of prostaglandin E1 was discountinued and immediately patient’s condition worsened. Subsequent treatment included re-administration of prostaglandin E1, iNO and mechanical ventilation. Both patients presented with transient improvement after application of iNO, but died despite maximized therapy. They were histopathologically diagnosed post-mortem with ACD. Array comparative genomic hybridization in patient one and patient two revealed copy-number variant (CNV) deletions, respectively, ~ 1.45 Mb in size involving *FOXF1* and an ~ 0.7 Mb in size involving *FOXF1* enhancer and leaving *FOXF1* intact.

**Conclusions:**

Both patients presented with a distinct course of ACD, extra-pulmonary manifestations and response to medications. Surgery and ceasing of prostaglandin E1 infusion should be considered as potential causes of this variability. We further highlight the necessity of thorough genetic testing and histopathological examination and propose immunostaining for CD31 and CD34 to facilitate the diagnostic process for better management of infants with ACD.

## Background

Respiratory failure is a common problem in the Neonatal Intensive Care Unit (NICU) and the diagnosis is often a challenge. Severe respiratory failure is usually observed in neonates with interstitial parenchymal lung diseases and congenital cardiac defects.

Alveolar capillary dysplasia with misalignment of pulmonary veins (ACDMPV) (MIM# 265380), frequently referred to as alveolar capillary dysplasia (ACD), is a rare disorder, leading to early severe respiratory distress with a persistent pulmonary hypertension (PPHN) and almost universally to death. Most of the affected infants present hypoxic respiratory failure within 48 h of life that is refractory to all medical therapies, including pulmonary vasodilators [1].

In 80–90% of histopathologically-verified ACD cases, loss-of-function of the *FOXF1* gene (MIM# 601089) on 16q24.1 or its distant upstream lung-specific enhancer has been described [1–4]. *FOXF1* encodes a Forkhead box F1 transcription factor primarily expressed in mesoderm-derived tissues during lung organogenesis, involved in development of pulmonary alveoli and capillaries [5–7].

Here, we present two neonates hospitalized in the tertiary NICU due to severe respiratory failure and pulmonary hypertension. Both patients were diagnosed with ACD due to different-sized heterozygous copy-number variant (CNV) deletions within the *FOXF1* gene locus on 16q24.1. They are the first patients with histopathologically- and genetically-confirmed ACD in Poland.

## Case presentation

### Case 1

A male neonate (birth weight: 2920 g) was born via caesarean section at 39+1 weeks of gestation in a non-tertiary hospital. On prenatal examination polyhydramnios, omphalocele, hydronephrosis of the right kidney and ventricular septal defect (VSD) were suspected. Amniocentesis showed 47, XY,+22 cells in the sample, however, postnatal karyotype was normal (46,XY). The Apgar scores were 9 at the 1^st^ and 10 at the 5^th^ minute of life. The infant was transferred to the NICU in a stable condition. In the 12^th^ hour of life, oxygen saturation measured with pulse oximetry (SpO_2_) decreased below 90% - passive oxygen therapy was applied with FiO_2_=0.6. Surgical repair of omphalocele was performed at 17^th^ hour of life. Due to respiratory deterioration after the surgery, the newborn received conventional mechanical ventilation with FiO_2_=0.4. Persistent pulmonary hypertension, atrial septal defect (ASD) and VSD were diagnosed on echocardiography examination. PPHN was treated with iNO but only with transient improvement. On the second day of life, the patient required ventilation with FiO_2_=1, surfactant administration and inotropes. On the third day, SpO_2_ remained persistently below 80%. Consequently, the infant was switched to high frequency ventilation but without any improvement. Despite continuation of this therapy during the next few days, SpO_2_ decreased further below 60%. Physical examination showed hepatosplenomegaly. Additional testing revealed coagulopathy, lack of peristalsis, congestion in the lungs, and metabolic acidosis. The patient died on the 13^th^ day of life after a cardiac arrest and ineffective cardiopulmonary resuscitation.

### Case 2

A male neonate (birth weight: 2400 g) was born via vaginal delivery at 39 weeks of gestation. On the prenatal examination fetal growth restriction and coarctation of the aorta (CoA) were suspected. The infant was born in a non-tertiary hospital in good condition; the Apgar scores were 8 at the 1^st^ and 10 at the 5^th^ minute. The newborn presented with peripheral cyanosis in the first hours of life and a difference between pre- and post-ductal saturation ranging about 10% was noted. Continuous infusion of prostaglandin E1 was applied due to prenatal suspicion of CoA. Initial echocardiogram showed ASD and VSD. On the second day of life, the infant was transferred to the NICU in Poznań. During the echocardiography examination infusion of prostaglandin was stopped and the infant suddenly deteriorated. CoA was not confirmed. The echocardiography revealed PPHN with a large ductus arteriosus (bidirectional shunt with right to left predominance) and narrow pulmonary veins. The patient was intubated, prostaglandin infusion was re-administered and PPHN was treated with iNO, resulting in immediate improvement. During the next few days, deterioration of the general condition with increasing oxygen demand up to 100% oxygen was observed. On the 9^th^ day of life, SpO_2_ was below 65% despite maximized respiratory support. Physical examination displayed liver and spleen enlargement. Additional testing showed hypotension, lack of peristalsis, congestion in the lungs and metabolic acidosis. On the 10^th^ day of life, cardiopulmonary resuscitation was not effective and the patient died due to cardiac arrest.

### Histopathological and genetic findings

#### Subjects

Lung tissue specimens were collected by post-mortem biopsy in both patients. Informed consents for histopathological examination of the lung samples and genetic testing were obtained from the patients’ parents. Lung tissue from a 2-week-old term infant was used as an internal control.

#### Histopathological evaluation

Formalin-fixed paraffin-embedded (FFPE) lung tissue samples were stained with hematoxylin and eosin (H&E). In addition, immunohistochemical (IHC) staining was performed on 4.5 μm FFPE tissue sections, using the En Vision™ FLEX GV800 (Dako) IHC Kit with specific monoclonal antibodies for CD34 and CD31. The IHC reaction was carried out using the OMNIS (Dako) or the BenchMark Ultra (Ventana Roche). Results were verified with an Olympus microscope. All stained tissue slides were archived by the VisionTek® Digital Microscope (Sakura).

#### DNA extraction

Genomic DNA isolation was performed using the MagCoreNucleid Acid Extractor (RBC Bioscience) with the sets of reagents dedicated to FFPE samples. DNA was quantified with the NanoDrop 2000 spectrophotometer (ThermoFisher Scientific).

#### Sanger sequencing

PCR reactions were performed using C1000 Touch thermal cycler (BioRad), with set of primers covering the whole coding region of *FOXF1*. Primers and PCR conditions are available on request. After amplification, PCR products were purified in an enzymatic reaction, diluted and sequenced using the forward and reverse primers with the BigDye Terminator 3.1 kit (ThermoFisher Scientific) according to manufacturer’s instruction. Next, products were purified by ethanol precipitation and analyzed with the 3500 Genetic Analyzer (Applied Biosystems) and the CodonCodeAlligner Software (CodonCode Corporation).

#### Array comparative genomic hybridization (array CGH)

The array CGH analysis of patients was performed using a customized 16q24.1-specific high-resolution 180 K microarray (Agilent Technologies), as described [3].

## Results

**Fig. 1:**
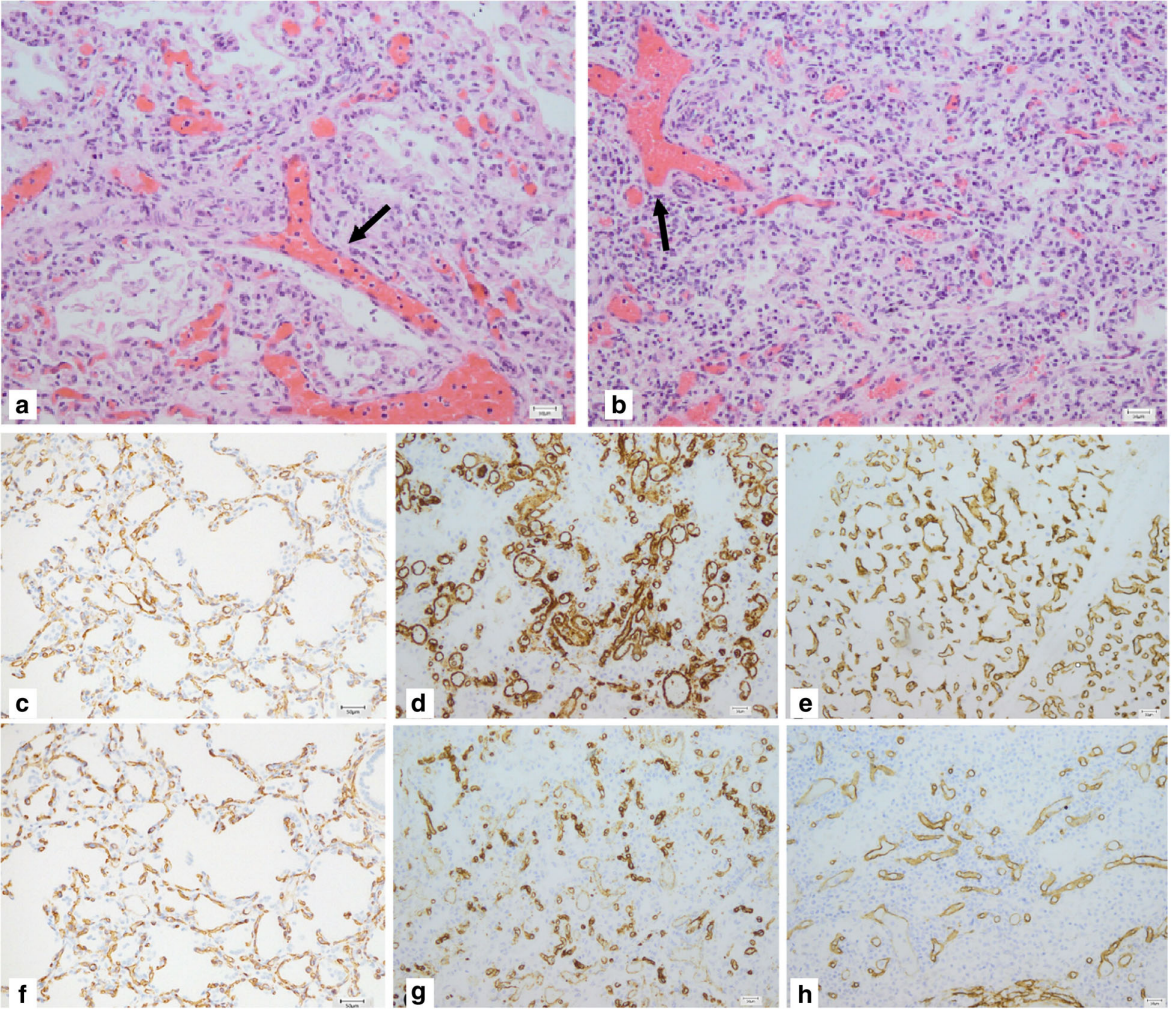
Histopathological examination of lung samples showing characteristic features of ACDMPV. Haematoxylin and eosin staining (H&E) of lung tissue obtained post-mortem showing abnormal shunt vessels (arrows) in the bronchovascular bundle and diffuse thickening of inter-alveolar septa with reduced density of capillaries. Immunostaining for the endothelial markers CD 31 and CD34 highlights poor approximation of the alveolar capillaries to epithelial cells and marked congestion compared to control lung from a term infant. As a result, the air-blood barrier was disrupted. H&E: **a**) patient 1, **b**) patient 2; CD31: **c**) control, **d**) patient 1, **e**) patient 2; CD34: **f**) control, **g**) patient 1, **h**) patient 2

In both cases, histopathological examination of post mortem lung biopsy samples revealed a significant decrease in the capillary network as well as abnormal shunt vessels in the bronchovascular bundle and blood-air barrier underdevelopment, features characteristic for ACD. Diffuse thickening of interalveolar septa, reduction of density and malpositioning of pulmonary alveolar capillaries were also observed. Residual acute alveolar damage was slightly more pronounced in Patient 1′ samples. Immunostaining for the endothelial markers CD34 and CD31 highlights poor approximation of the alveolar capillaries to epithelial cells and marked congestion compared to control lung from the term infant resulting in disruption of air-blood barrier (Fig. 1.)

**Fig. 2:**
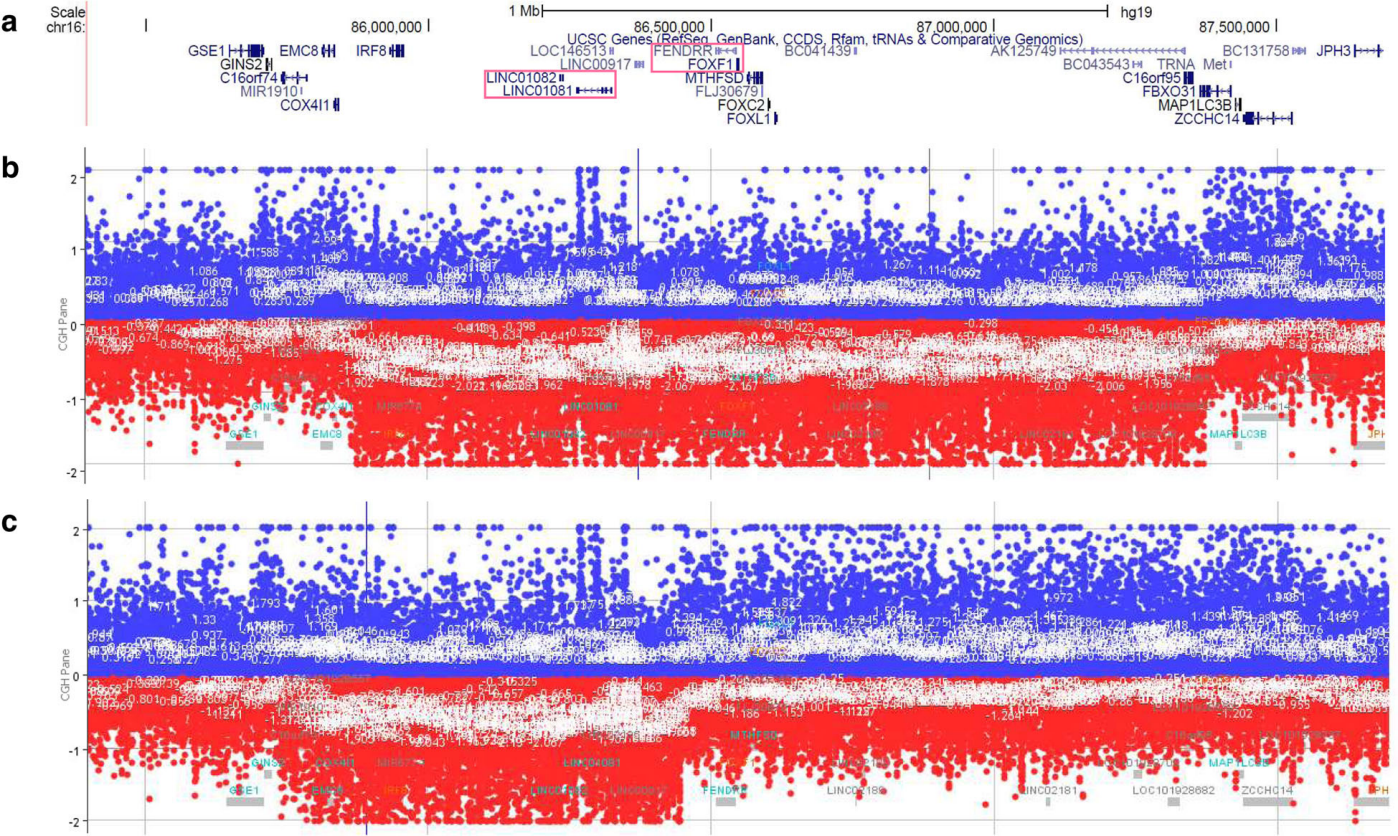
Results of array CGH analyses. **a** Schematic representation of genes mapping within the 16q24.1 (hg19) interval, including *FOXF1*, *FENDRR*, *LINC01081* and *LINC01082 *(pink rectangles). **b** Array CGH plot showing an ~ 1.45 Mb CNV deletion in Patient 1, involving the *FOXF1* gene, its upstream enhancer (*LINC01082*, *LINC01082*), and *IRF8*, *LINC00917*, *FENDRR*, *MTHFSD*, *FOXC2*, and *FOXL1*. **c** Array CGH plot showing an ~ 0.7 Mb CNV deletion in Patient 2, involving the upstream *FOXF1* enhancer (*LINC01082* and *LINC01082*), *COX411*, *IRF8*, *LINC00917*, and leaving the *FENDRR* and *FOXF1 *genes intact

Direct sequencing did not reveal any clinically relevant single nucleotide variants or indels within the coding portion of *FOXF1* in these patients. Array CGH revealed the heterozygous CNV deletions at 16q24.1 region in both cases. In Patient 1, an ~ 1.45 Mb CNV deletion (chr16:85,863,000-87,370,500, hg19) involving *FOXF1,* its upstream enhancer (*LINC01082*, *LINC01082*) and *IRF8*, *LINC00917*, *FENDRR*, *MTHFSD*, *FOXC2* and *FOXL1* was identified (Fig. 2b). In the second patient, an ~ 0.7 Mb CNV deletion (chr16:85,738,000-86,446,500, hg19) removed the upstream *FOXF1* enhancer (*LINC01082* and *LINC01082*) and *COX411*, *IRF8*, *LINC00917*, leaving the *FOXF1* gene intact (Fig. 2c). The inheritance status of these deletions is unknown.

## Discussion and conclusions

ACD is a rare lethal lung developmental disorder characterized by severe pulmonary hypertension observed typically shortly after birth [1]. Histopathologically, disruptions in lung development result in a reduced density of lung vessels and abnormal lobular structure. Currently, there is no successful treatment available for patients diagnosed with the typical presentation of ACD. Neither concomitant administration of iNO and prostacyclin nor the use of paracorporeal lung assist device followed by lung transplantation were effective [8]. However, a few infants with atypical ACD characterized by late onset of symptoms, milder manifestations or focal histopathological changes responded to therapy lasting for months [9–11]. Bilateral lung transplant may be a therapeutic alternative in these patients; its effectiveness was similar to lung transplants performed in patients with other indications [12]. Of note, all the infants reported with successful lung transplant underwent open lung biopsy before the procedure and the diagnosis was known prior to the surgery [1, 12] Hence, early diagnosis of ACD is vital in the decision-making process.

Thus far, only one case of an ACD newborn was reported in Poland. However, no genetic testing was performed [13]. Here, we present two cases of ACD that were confirmed by both histochemical and genetic testing. Different-sized heterozygous losses at chromosome 16q24.1 were detected using array CGH analyses. Whereas the CNV deletion in Patient 1 involved *FOXF1*, the CNV deletion observed in Patient 2 removed the upstream *FOXF1* enhancer, leaving the *FOXF1* gene intact. These findings further confirm that the non-coding genomic interval mapping upstream to *FOXF1* is essential for human lung development [14].

Unfortunately, genetic testing is not always easily available. Moreover, the negative genetic result does not exclude the diagnosis in suspected patients [15] and histopathological assessment of lung biopsy or autopsy samples by an experienced pathologist remains the gold standard in diagnosing ACD. Recommendations of the chILD Pathologic Co-operative Group state that apart from standard H&E stains, appropriate staining for structural components and immunostaining should be adjusted to the clinical situation [16]. However, there are no specific guidelines which methods are the most useful diagnostic tools. Since CD31 is the most specific and sensitive marker visible in paraffin sections showing both small and large vessels and CD34 determines microvessels density, we propose that immunostaining for these markers can bring added value in the ACD diagnostic process.

It is typical for patients diagnosed with ACD to present with good general condition within first 24–48 h of life, known also as a “honeymoon period”. However, Patient 1 required a high fraction of oxygen already 12 h after birth and the asymptomatic period was exceptionally short. The course of PPHN was fulminant without noticeable effects of the therapy. On the contrary, Patient 2’s condition worsened only on the 3^rd^ day of life exceeding the typical asymptomatic stage. Moreover, his health status further deteriorated gradually after the initial response to iNO. Interestingly, prompt presentation of ACD symptoms in this case was triggered by discontinuance of the intravenous infusion of prostaglandin E_1_. The mechanism of this deterioration is probably best explained by right ventricular (RV) failure. As the PDA got narrower or closed after ceasing prostaglandin E_1_, RV failure worsened from pumping against high pulmonary vascular resistance associated with ACD. Given that occasionally patients are asymptomatic within the neonatal period, potential triggers leading to pulmonary hypertensive crisis and fatal exacerbation should be thoroughly investigated. Goel et al. suggested the possible factors can include respiratory or urinary tract infections [17]. In our patients, clinical conditions deteriorated after experienced stress either associated with surgery or with rapid discontinuation of prostaglandin infusion, suggesting that these factors could play a role in the origin of ACD symptoms.

In histopathological examination both neonates had the similar lung defect. Patient 1 had more alveolar damage, however it may be due to longer mechanical ventilation that was initiated sooner during the treatment process. Although most authors claim that there are no visible differences in histopathologic images between patients with fulminant form of the disease presented in neonatal period and so-called “long survivors”, some argue that the abnormalities in the tissue architecture, especially capillary density, lobular development and range of involvement are less severe among children with late onset of ACD [17]. Moreover, Melly et al. described a phenotype characterized by overlapping ACD and chronic lung disease features, based on clinical data and histopathologic findings [18]. They highlighted older age at diagnosis, partially better outcome and higher density of capillaries seen in the lung material taken from these patients as compared to children recognized as typical ACD. It may constitute a step forward distinguishing prediction factors of survival and relatively better clinical course among affected children.

Previous results showed that haploinsufficiency of *FOXF1* either due to point mutations or CNV deletions overlapping *FOXF1* or its upstream regulatory region leads to full lung manifestation of ACD [3]. Only once, the 16q deletion involving *FOXF1* enhancer was associated with pulmonary capillary hemangiomatosis [19]. In contrast, phenotypic differences have been observed for co-existing extra-pulmonary anomalies. Interestingly, hypoplastic left heart syndrome (HLHS) and single umbilical artery have been reported in ACD newborns with CNV deletions involving *FOXF1, FOXC2, FOXL1*, and *FENDRR* [3]; only one infant with ACD and HLHS caused by de novo likely pathogenic c.209_214del (p.Thr70_Leu71del) variant in *FOXF1* was described [20]. However, screening of *FOXC2* and *FOXL1* in patients with HLHS revealed no point mutations in those genes [21]. Of note, homozygous deletion of *Fendrr* in mice results in lethal cardiac and lung defects [22], suggesting that heart anomalies observed in ACD patients can be associated with *FENDRR* disruption.

While both newborns, reported here, were diagnosed with heart defects, ASD and VSD, Patient 1 also presented other extra-pulmonary features, including omphalocele, hydronephrosis, and polyhydramnios observed in prenatal examination. Except for omphalocele, similar non-lung manifestation was reported by Yu et al. in another infant with ACD and overlapping CNV deletion on 16q24, including *FENDRR* and *FOXF1* [23]. In addition, the coexistence of ACD with CoA and hydronephrosis was observed in the newborn with ACD (pt 135.3) and similar CNV deletion [3]. On the other hand, Patient 2 had 16q24 CNV deletion similar to deletion previously detected in pt. 47.4 (D9), which involved the upstream enhancer region, overlapping long non-coding RNA genes*, LINC01081* and *LINC01082* and leaving *FENDRR* and *FOXF1* intact [3, 24]. In contrast to pt. 47.4 (D9) [3, 24], who had intestinal malrotation, imperforate anus, and butterfly vertebrae, no similar defect was observed in our Patient 2.

Most of CNV deletions found in patients with ACD arise de novo on the maternal chromosome, what suggests genomic imprinting of the *FOXF1* locus [3]. However, in the described cases the inheritance status of these deletions is unknown as samples from the parents were not available for analysis.

In conclusion, we present two cases diagnosed with ACD based on typical histopathological picture but with diverse clinical presentation and distinct abnormalities within the *FOXF1* gene cluster on 16q24.1. Presented cases emphasize the importance of both genetic testing and histopathological examination of the lung in neonates with refractory respiratory failure.

## Data Availability

The datasets used and/or analysed during the current study are available from the corresponding author on reasonable request.

## Abbreviations

ACD: Alveolar capillary dysplasia
ACDMPV: Alveolar capillary dysplasia with misalignment of pulmonary veins
array CGH: array comparative genomic hybridization
ASD: Atrial septal defect
CLD: Chronic lung disease
CNV: Copy-number variant
CoA: Coarctation of the aorta
FFPE: Formalin-fixed paraffin-embedded
H&E: Hematoxylin and eosin
HLHS: Hypoplastic left heart syndrome
IHC: Immunohistochemical
iNO: inhaled nitric oxide
NICU: Neonatal Intensive Care Unit
PDA: Patent ductus arteriosus
PPHN: Persistent pulmonary hypertension of a newborn
RV: Right ventricle
SpO_2_: Oxygen saturation measured by pulse oximetry
VSD: Ventricular septal defect
